# Interspecific Interactions Drive Nonribosomal Peptide Production in Nodularia spumigena

**DOI:** 10.1128/aem.00966-22

**Published:** 2022-07-12

**Authors:** Sandra Lage, Hanna Mazur-Marzec, Elena Gorokhova

**Affiliations:** a Department of Environmental Science, Stockholm University, Stockholm, Sweden; b Division of Marine Biotechnology, Institute of Oceanography, University of Gdańsk, Gdańsk, Poland; Georgia Institute of Technology

**Keywords:** Baltic Sea, allelopathy, coculture, anabaenopeptins, spumigins, nodularins

## Abstract

Nodularia spumigena is a bloom-forming cyanobacterium that produces several classes of nonribosomal peptides (NRPs) that are biologically active; however, the ecological roles of specific NRPs remain largely unknown. Here, we explored the involvement of NRPs produced by N. spumigena in interspecific interactions by coculturing the cyanobacterium and its algal competitors, the diatom Phaeodactylum tricornutum and the cryptomonad Rhodomonas salina, and measuring NRP levels and growth responses in all three species. Contrary to the expected growth suppression in the algae, it was N. spumigena that was adversely affected by the diatom, while the cryptomonad had no effect. Reciprocal effects of N. spumigena on the algae were manifested as the prolonged lag phase in R. salina and growth stimulation in P. tricornutum; however, these responses were largely attributed to elevated pH and not to specific NRPs. Nevertheless, the NRP levels in the cocultures were significantly higher than in the monocultures, with an up to 5-fold upregulation of cell-bound nodularins and exudation of nodularin and anabaenopeptin. Thus, chemically mediated interspecific interactions can promote NRP production and release by cyanobacteria, resulting in increased input of these compounds into the water.

**IMPORTANCE** NRPs were involved in growth responses of both cyanobacteria and algae; however, the primary driver of the growth trajectories was high pH induced by N. spumigena. Thus, the pH-mediated inhibition of eukaryotic phytoplankton may be involved in the bloom formation of N. spumigena. We also report, for the first time, the reciprocal growth inhibition of N. spumigena by diatoms resistant to alkaline conditions. As all species in this study can co-occur in the Baltic Sea during summer, these findings are highly relevant for understanding ecological interactions in planktonic communities in this and other systems experiencing regular cyanobacteria blooms.

## INTRODUCTION

In the brackish Baltic Sea, the summer bloom of filamentous, diazotrophic cyanobacteria is a recurring natural phenomenon (1). These blooms consist of Nodularia spumigena, *Aphanizomenon* spp., and, to a lesser extent, *Dolichospermum* spp. (formerly *Anabaena* spp.) (2). Due to their ability to fix atmospheric nitrogen, these diazotrophic cyanobacteria can outcompete eukaryotic algae and dominate the phytoplankton community, especially at low nitrogen-to-phosphorus ratios (3). The ongoing climate change and eutrophication facilitate the bloom frequency and intensity (4, 5), even though the environmental preferences among the species contributing to these blooms vary.

Cyanobacteria produce many toxins and bioactive substances, many of which are peptides (6). In addition to the well-studied hepatotoxic nodularins (NODs), N. spumigena produces four other classes of nonribosomal peptides (NRPs), i.e., spumigins (SPUs), aeruginosins (AERs), pseudoaeruginosins, and anabaenopeptins (APs), and many other unknown bioactive metabolites (7, 8). It has been suggested that NRPs, including nodularins, help cyanobacteria to survive under fluctuating biotic and abiotic conditions (9–12); however, their functions remain largely unknown. Any of the metabolites produced by cyanobacteria may alter cyanobacteria bloom formation and maintenance by acting as autoinducers/inhibitors and, due to allelochemical properties, affect competitors (13, 14). It has been suggested that exudates of Baltic N. spumigena have such allelochemical properties toward heterotrophic bacteria, eukaryotic algae, and other cyanobacteria; however, both inhibiting and stimulating effects on potential competitors as well as no-effect outcomes have been reported (15–21). For instance, in cocultured N. spumigena and Rhodomonas salina, a reduction of the cryptomonad growth rate was reported (20), whereas coculturing of the cyanobacterium with P. tricornutum did not affect the diatom growth (19). Moreover, the effects appear to be strain specific because when other strains of N. spumigena and R. salina were used, no significant differences in the cryptomonad growth were observed, albeit there was a reduction in the population-carrying capacity of the cryptomonad (19, 20).

Nodularins were first suggested to be responsible for N. spumigena allelopathic activity; however, no correlation between nodularin production and allelopathic effects has been found (16, 21). Notably, the adverse effects of N. spumigena on eukaryotic algae have been suggested to be controlled by the pH increase in cyanobacteria cultures and not by the allelochemicals produced (19). Reciprocally, eukaryotic algae (e.g., the green algae Tetraselmis suecica [20]) and other cyanobacteria (*Aphanizomenon* spp. [22, 23]) were found to inhibit N. spumigena growth. Moreover, N. spumigena exposed to cell-free filtrate of the picocyanobacterium *Synechococcus* spp. decreased in abundance with no significant photosynthetic efficiency response (24). The growth inhibition in N. spumigena was exacerbated by coculturing with *Synechococcus* spp., accompanied by a decrease in chlorophyll *a* (Chl*a*) and carotenoid content, misshapen cells, and cell lysis (25). Therefore, reciprocal allelopathic responses between the cyanobacterium and other phytoplankton species (both eukaryotic algae and picocyanobacteria) are expected, but only a few studies provided quantitative data. Knowing how NRPs regulate phytoplankton assemblages could help understand taxonomic and functional succession in plankton, bloom initiation and progression, and effects on pelagic food webs.

Here, we investigated whether NRPs produced by the Baltic Sea N. spumigena in cocultures with potential competitors are involved in chemically mediated interspecific interactions. We hypothesized that reciprocal interactions would occur in cocultures of N. spumigena with eukaryotic algae, with more substantial impacts on the competitors than on the cyanobacterium due to the NRP production (hypothesis 1 [H1]). Further, we hypothesized that NRPs involved in growth inhibition (and evidenced by a negative relationship between the competitor growth and NRP concentrations) would be detected extracellularly in the cocultures. Moreover, the total concentrations of the NRPs involved in allelopathic interactions will be higher in the cocultures than in the N. spumigena monoculture (H2). Furthermore, should the cyanobacterium-induced pH variation drive the effects as previously suggested (19), a pH increase in the cocultures would coincide with concomitant growth inhibition in the competitor (H3). Finally, as NRP production has been associated with optimal growth of the cyanobacteria (26, 27), we expected to find positive relationships between the total NRP concentrations and N. spumigena growth (H4).

To investigate chemically mediated interactions, coculture experiments designed to physically separate test species but allow diffusion of metabolites are advocated (28, 29). Here, we used a coculture system with N. spumigena and each test competitor incubated in individual chambers separated by a membrane filter (see Fig. S1 in the supplemental material). This system simulated a co-occurrence of these species in *Pelagia* and allowed us to study interspecific interactions caused by diffusible metabolites passing through the membrane without direct cell-to-cell contact (11). The monocultures were grown in the same manner as the cocultures but with the same species in both chambers. We studied chemically mediated interactions between N. spumigena and two potential competitors, the diatom Phaeodactylum tricornutum and the cryptomonad Rhodomonas salina. These algae co-occur with N. spumigena in the Baltic Sea and have differing susceptibilities to the cyanobacterium, as suggested by earlier studies, i.e., no effect and growth inhibition, respectively (19, 20). To our knowledge, this is the first study examining the involvement of NRPs other than nodularin in interspecific interactions with N. spumigena.

## RESULTS

### Growth responses to the coculture.

**FIG 1:**
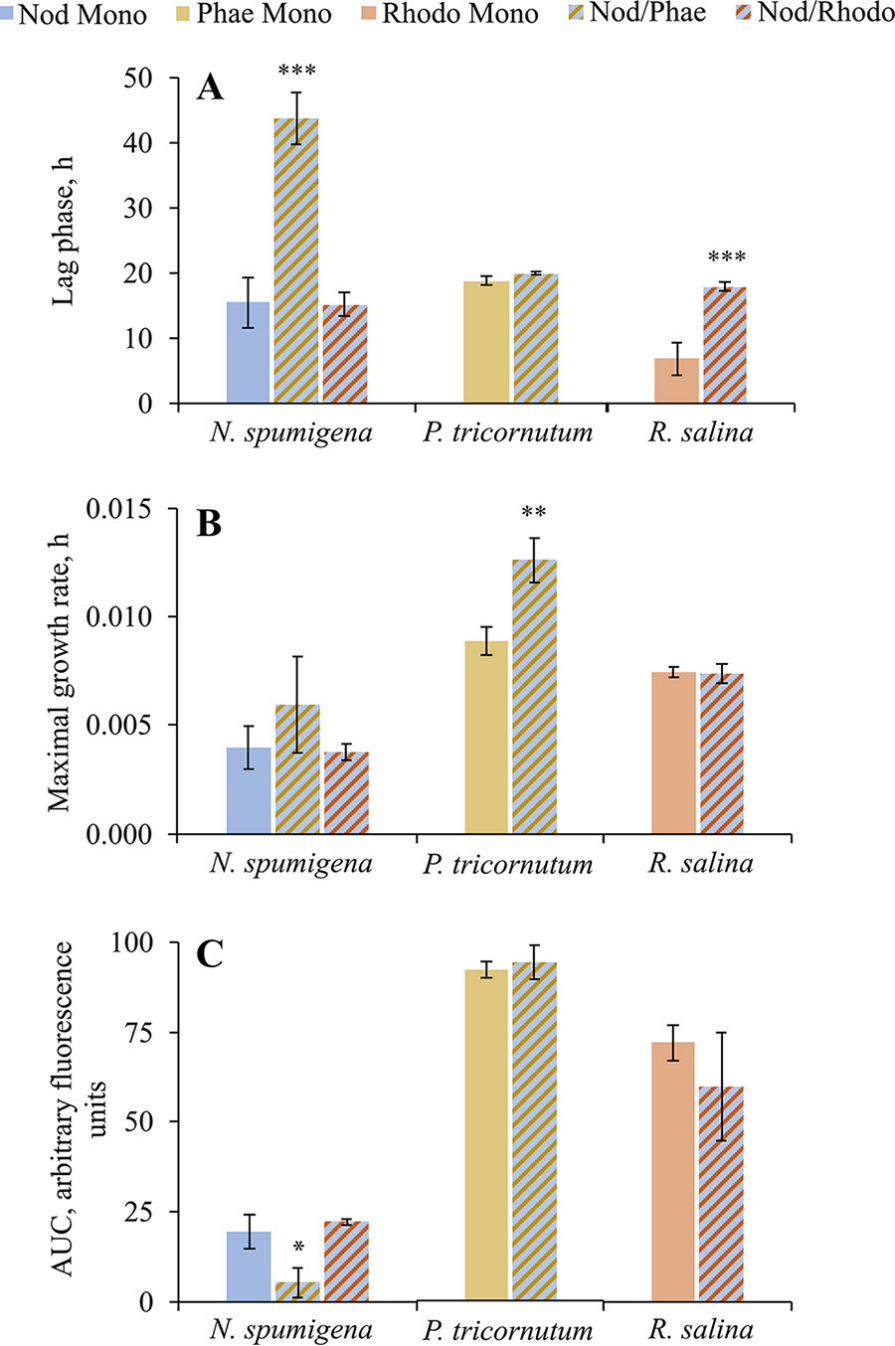
Growth responses of N. spumigena (Nod), P. tricornutum (Phae), and R. salina (Rhodo) under monoculture (mono) and coculture treatments (mean and SE; *n *= 3). (A) Lag phase preceding the active growth (λ, h). (B) Maximal growth rate estimated for the exponential period (μ, d^−1^). (C) Area under the curve (AUC) representing algal production during the exposure. Monocultures are shown with solid colors and cocultures are shown in stripe patterns. Asterisks indicate a significant difference from the monoculture; *, *P < *0.05; **, *P < *0.01; and ***, *P < *0.001 (GLM; Table S3 in the supplemental material).

Positive growth was observed in all species and treatments (Fig. 1B). The growth of N. spumigena and R. salina was adversely affected in the cocultures N. spumigena/*P. tricornutum* and N. spumigena/R. salina, respectively, as indicated by significantly higher lag-phase duration than in the respective monocultures (H1; λ values) (Fig. 1A and see Table S3 in the supplemental material). The maximal growth rate of P. tricornutum was significantly higher in the coculture with N. spumigena (N. spumigena/P. tricornutum) than in the respective monoculture (Fig. 1B and Table S3), with a concomitant decrease in the N. spumigena area under the curve (AUC) values (Fig. 1C and Table S3).

There was a significant positive relationship between the maximal growth rate and lag-phase duration in N. spumigena monoculture and N. spumigena/R. salina; no significant relationship was found in N. spumigena/P. tricornutum (H1; Fig. S4 and Table S4). The growth trajectory of N. spumigena was similar between the monoculture and N. spumigena/R. salina (Fig. S4), whereas in N. spumigena/P. tricornutum, a prolonged (>2-fold) lag phase was observed, suggesting that the N. spumigena/P. tricornutum growth curve deviated from the other two treatments. However, given the low number of replicates and a rather high variability within the N. spumigena/P. tricornutum treatment, the result is not conclusive. For R. salina and P. tricornutum, a positive relationship between the maximal growth rate and lag-phase duration was observed in the monocultures (only significant in P. tricornutum), while the relationships were not significant in the cocultures (H1; Table S4).

### Cell-bound and extracellular NRPs in mono- and cocultures.

In total, 11 cell-bound NRPs were detected in N. spumigena mono- and cocultures (Fig. S5A). No extracellular NRPs were detected in the N. spumigena monoculture (Fig. S5A), whereas two extracellular NRPs were detected in the cocultures N. spumigena/P. tricornutum and N. spumigena/R. salina (H2, Fig. S5A). Some NRPs might have been present extracellularly, but their concentrations were below the limit of detection.

**FIG 2:**
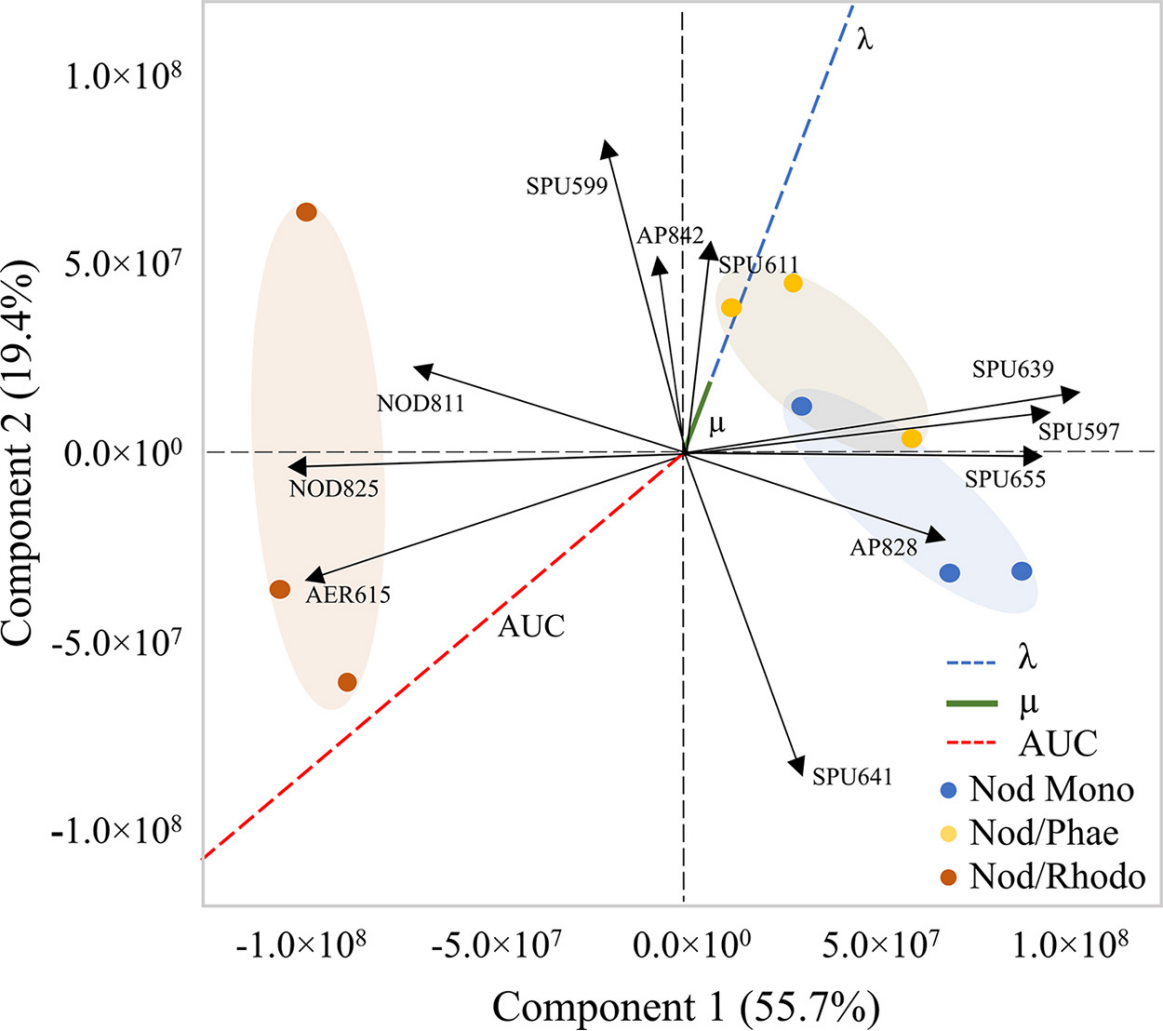
Principal-component analysis (PCA) on covariances of the sum of cell-bound and extracellular NRPs of N. spumigena monocultures (blue) and cocultures P. tricornutum (yellow) and R. salina (orange). The growth parameters lag phase (λ, h), maximal growth rate (μ, d^−1^), and area under the curve (AUC) were used as supplementary variables; these parameters are shown by dashed blue, solid green, and dashed red lines, respectively. NRPs are denoted as SPU, spumigin; NOD, nodularin; AER, aeruginosin; and AP, anabaenopeptin.

The ordination of the total NRP levels in the N. spumigena mono- and cocultures resulted in a single significant PC (Fig. S4) that explained about 56% of the NRP variance (Table S2). A clear separation was observed in the principal-component analysis (PCA) biplot between N. spumigena monoculture and N. spumigena/R. salina, whereas there was some overlap between N. spumigena monoculture and N. spumigena/P. tricornutum (Fig. 2). The spumigins (SPU 597 and SPU 639) had the highest positive loadings (≥0.3), while the aeruginosin (AER 615) and the nodularins (NOD 811 and NOD 825) had the highest negative loadings on the PC1 and, hence, influenced the grouping (Fig. S3). Thus, the low SPU 597 and SPU 639 concentrations coincided with the high AER 615, NOD 811, and NOD 825 concentrations (i.e., in N. spumigena/R. salina).

**FIG 3:**
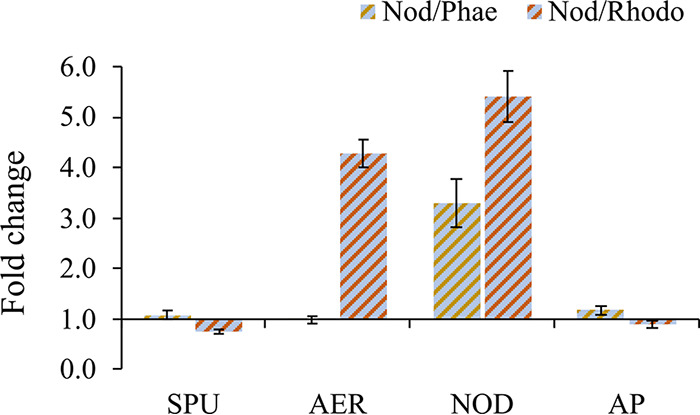
Relative concentrations of total (cell-bound and extracellular) NRPs pooled by class in the N. spumigena cocultures with P. tricornutum (Nod/Phae) and R. salina (Nod/Rhodo), given as a fold change over N. spumigena monoculture. NRP classes include SPU, spumigin; NOD, nodularin; AER, aeruginosin; and AP, anabaenopeptin. Fold change of 1 represents equilibrium, i.e., concentrations equal to monoculture.

Some total NRP concentrations differed significantly between the monoculture and the cocultures (H2; Fig. S5B and Table S5). Overall, N. spumigena had significantly higher total concentrations of nodularins in the cocultures than in the monoculture, with a fold change ranging from 3 to 5, depending on the coexposed species (H2; Fig. 3 and Table S5). In N. spumigena/R. salina, the total aeruginosins were significantly higher, whereas spumigins (i.e., SPU 597, SPU 639, and SPU 655) were significantly lower than in the monoculture (Fig. 3, Fig. S5B, and Table S5).

There was no significant relationship between the competitor growth and either the extracellular NRPs (NOD 825 and AP 842) or the NRPs that were upregulated in the cocultures but not detected extracellularly (SPU 599, AER 615, and NOD 811) (H2; Table S6). However, SPU 611 was significantly negatively related to P. tricornutum and positively to the growth of R. salina (Table S6). Moreover, there was a significant negative relation between AP 828 and the growth of R. salina (Table S6).

### pH and its effects on growth.

**FIG 4:**
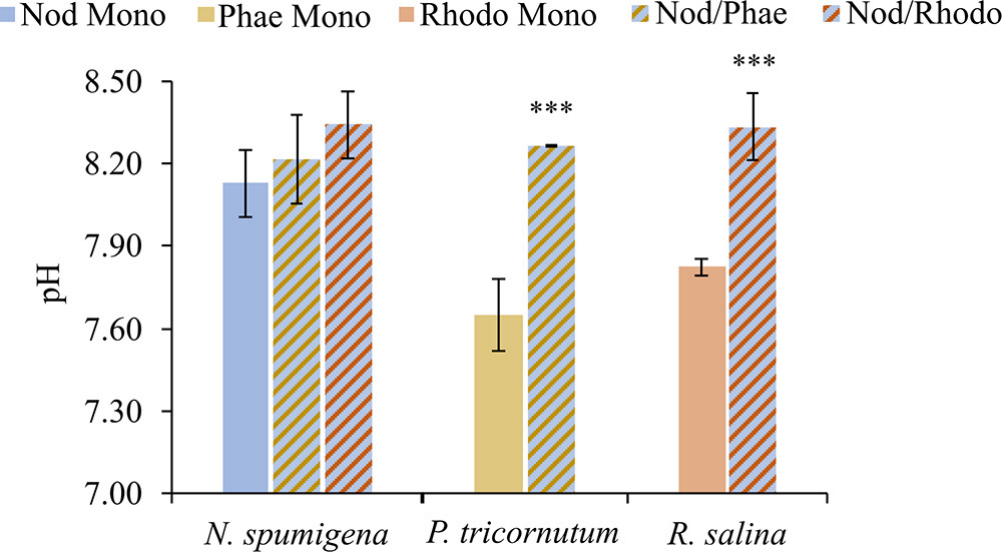
pH values in mono- and cocultures of N. spumigena (Nod), P. tricornutum (Phae), and R. salina (Rhodo). Monocultures are shown with solid colors, and cocultures are shown in stripe patterns. Asterisks indicate significant differences from the monoculture; *, *P < *0.05; **, *P < *0.01; and ***, *P < *0.001 (GLM; see Table S3 in the supplemental material).

In the cocultures and N. spumigena monoculture, a significantly higher pH was observed than in the algal monocultures (H3; Fig. 4 and Table S7). Furthermore, a prolonged lag phase in the N. spumigena/R. salina coculture was observed, with a significantly positive relationship between R. salina lag-phase duration and pH, indicating growth inhibition. On the contrary, a significant positive relationship at an alpha value of 0.1, between P. tricornutum maximal growth rate and pH (*P = *0.051) indicated a stimulatory effect on the diatom growth in the coculture (H3; Table S8). No other significant pH effects on the growth parameters in the test species were observed.

### Relationships between total NRPs and growth in N. spumigena.

**FIG 5:**
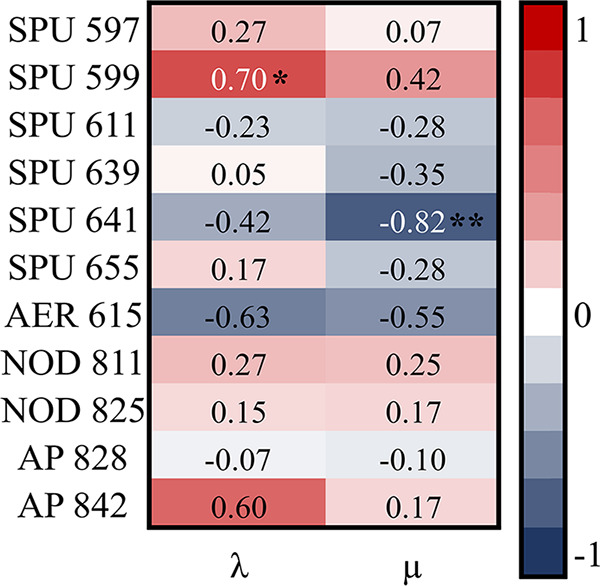
The Spearman rank correlations between the physiological parameters (λ, lag phase, and μ, maximal growth rate) and NRPs measured in the N. spumigena monoculture and cocultures with P. tricornutum and R. salina. The numbers in the cells are *rho* values; *, *P < *0.05, and **, *P < *0.01.

Production of spumigins SPU 599 and SPU 641 was significantly associated with a prolonged lag phase and decreased growth rate, respectively, in the cyanobacterium (Fig. 5). No significant correlations between the growth parameters and NRPs (nodularin, aeruginosin, and anabaenopeptins) were found. Significant correlations at alpha values of 0.1 between the lag phase and AP 842 (positive; *P = *0.08) and between lag phase and AER 615 (negative; *P = *0.06) were noted.

## DISCUSSION

### N. spumigena growth was inhibited by P. tricornutum but not by R. salina.

We expected to observe reciprocal interactions between the cyanobacterium and its potential competitors, with more substantial net effects on the competitors than the cyanobacterium (H1). However, contrary to our hypothesis, the most pronounced effect was the suppression of N. spumigena growth by P. tricornutum (Fig. 1A; see Table S3 in the supplemental material). The prolonged lag phase in N. spumigena observed in the N. spumigena/P. tricornutum coculture with no compensatory growth (Table S4 and Fig. S4) resulted in a significant production loss in the cyanobacterium (Fig. 1C and Table S3, AUC values). Assuming that a positive relationship between the lag phase and maximal growth rate indicates adaptation to the test conditions (30, 31), N. spumigena has demonstrated a limited capacity to adapt and compete with P. tricornutum in our experimental system.

To our knowledge, this is the first report of N. spumigena growth inhibition by diatoms. In most studies examining cyanobacteria allelopathy toward diatoms, cryptomonads, and other eukaryotic algae, the extracts/cell-free filtrates of the cyanobacterium were added to the alga monoculture, thus not allowing detection of reciprocal effects (15–18, 32, 33). However, growth inhibition in algae and bacteria (29, 34) induced by diatoms has been observed, including P. tricornutum that produces allelochemicals with negative effects on the raphidophyte Heterosigma akashiwo, the dinoflagellate Prorocentrum donghaiense, the green algae Dunaliella salina, and the coccolithophore Emiliania huxleyi, as well as some bacteria (35, 36). Moreover, a potent allelochemical belonging to glycinamides (C_30_H_38_N_6_O_6_) has been isolated from P. tricornutum (35). Further biochemical studies on the nature of the bioactive compounds released by P. tricornutum are needed to understand their involvement in the growth inhibition of phytoplankton species.

No effects on the N. spumigena growth induced by R. salina and the similarity of the growth trajectories between N. spumigena/R. salina and the cyanobacterium monoculture (H1; Fig. 1 and Table S3) agree with previous studies with mixed cultures (20). However, it is possible that our growth assessment based on *in vivo* Chl*a* fluorescence measurements was not sufficiently sensitive to detect changes in cyanobacteria and algae biomass. Using cellular carbon quota and cell number could have provided a more robust biomass assessment (37, 38).

Moreover, considering that cultures were nonaxenic, the associated microbiome of the cyanobacteria and the eukaryotic algae might also contribute to these intraspecific interactions. It has been previously observed that associated bacteria influence algal abundance and performance as well as the excretion and/or metabolization of metabolites, which can, in turn, influence their chemically mediated interactions (29, 39, 40).

### Growth of eukaryotic competitors was affected by high pH.

As hypothesized (H3), pH was elevated in the N. spumigena monoculture and the cocultures (Fig. 4; Table S7), which is in line with reported pH increase during cyanobacteria blooms (41, 42). In the Baltic Sea, for example, pH >9.3 has been recorded in cyanobacteria microenvironments (43). Oligohaline waters have a lower pH-buffering capacity than seawater, which explains the pH rise due to cyanobacterial photosynthetic carbon fixation (44) and promotes cyanobacteria blooms, especially when inorganic N supply from sediment (45) and phosphorus solubility (46) increase. The elevated pH was associated with growth inhibition in R. salina and its stimulation in P. tricornutum (Table S8). Thus, not the allelopathy but the pH-mediated effects and tolerance to the high pH values can be the basis of N. spumigena outcompeting eukaryotic algae in bloom conditions (19).

While N. spumigena was not affected by R. salina, the latter had a weak, yet statistically significant, growth suppression in the coculture, indicated by the prolonged lag phase (Fig. 1A and Table S3). However, no significant decrease in biomass production was found (Fig. 1C and Table S3, AUC values). Thus, although statistically significant, the effect was not biologically meaningful. Under mixed-culture conditions N. spumigena did not affect R. salina growth rate, but the stationary phase of the cryptomonad was reached earlier in the mixed culture than in the monoculture, followed by decreased cell density (19). Growth was inhibited in *Rhodomonas* spp. (such as R. salina) exposed to either cell-free filtrates of N. spumigena (15) or in the mixed culture with the cyanobacterium. In these studies, the inhibition degree varied, most likely due to different experimental designs (i.e., cell filtrates, mixed culture, or coculture) and the culture pH (increased, unchanged, or not measured) (15, 19, 20). Also, different strains of N. spumigena and R. salina were used in these studies with possible implications for the cyanobacterium competitiveness and the cryptomonad susceptibility (47). In the previous studies, the N. spumigena strains KAC 13, KAC 66, and AV1 and the R. salina strains KAC 30, K-0294, and TV22 were used, while in the present study, we used N. spumigena CCNP1403 and R. salina CCAP 978/24 (Table S1) (15, 19, 20). Finally, different growth endpoints were used, with growth rate and cell abundance being the most common metrics reported, whereas the lag-phase duration was not measured in any of these previous studies. Notably, this was the most sensitive endpoint in our experiment.

The prolonged lag phase in R. salina was likely to be driven by the elevated pH (H3; Fig. 4; Table S7). Additional evidence for the high pH as the primary stressor for R. salina is the significant positive relationship between the lag-phase duration and culture pH, thus supporting hypothesis H3 (Table S8). However, as there was no significant change in the R. salina AUC values in the coculture (Fig. 1 and Table S3), it is plausible that once the alga became adapted to the higher pH during the lag phase, the growth resumed at the normal rate. In R. salina, a decrease in cell density has been observed at high (>10.5) pH levels (19), and recently, a growth inhibition at pH 8.5 compared to pH 7 has been demonstrated (48). In our experiment, however, the pH values never exceeded 8.5, which might explain the adaptation and compensatory growth resulting in no significant change in the AUC values. The successful adaptation to high pH values can also explain the observed coexistence of R. salina and N. spumigena in the Baltic Sea (49). However, the adaptive capacity to pH may vary among different strains of R. salina (47), which might also explain the decrease in cryptomonads in the Gulf of Finland during late 1970 that has been suggested, at least in part, to result from the increase in cyanobacteria (50).

Contrary to the hypothesized suppression by the cyanobacterium (H1) and high pH (8.3) in the coculture (H3), the diatom growth was stimulated by N. spumigena (Fig. 1B and Table S3). Moreover, a significant positive relationship at an alpha value of 0.1 between P. tricornutum maximum growth rate and culture pH (*P = *0.051; Table S8) was found. Previously, Møgelhøj et al. (19) found no significant effect of N. spumigena on P. tricornutum growth under mixed-culture conditions and suggested that P. tricornutum has a high capacity to grow well over a wide pH range (19, 51), which is supported by our results, even though we used different strains of N. spumigena and P. tricornutum. However, the combined effect of pH and NRP produced by the cyanobacterium on the diatom growth stimulation cannot be ruled out.

### Interspecific chemically mediated interactions alter NRPs concentrations of N. spumigena.

We expected to find higher total NRP concentrations in the cocultures than N. spumigena monoculture (H2); however, coculturing induced both upregulation and downregulation of NRPs (Fig. 3 and Fig. 5). The NOD class, for example, was up to 5-fold higher in the cocultures than in the monoculture (H2; Fig. 3 and Fig. 5). Despite no or weak growth responses of R. salina to the cyanobacterium, total concentrations of spumigins (SPU 597, SPU 639, and SPU 655) were significantly lower, and AER 615, NOD 811, NOD 825, and AP 842 were significantly higher in the coculture than in the N. spumigena monoculture (H2; Fig. S6 and Table S5). In the coculture with P. tricornutum, where inhibition of N. spumigena (Fig. 1) and stimulation of P. tricornutum were observed, significantly higher total SPU 599, NOD 811, and AP 842 concentrations were found than in the N. spumigena monoculture, and no NRP was significantly lower than in the cyanobacterium monoculture (H3; Fig. S5 and Table S5). The upregulation of NRPs in the cocultures is in line with *Microcystis* responses, where the metabolite production usually increases when other cyanobacteria are introduced into the system (12, 52). For example, in Microcystis aeruginosa cocultured with Planktothrix agardhii, a 5-fold increase in intracellular metabolites, including 390 compounds and 5 targeted peptides (cyanopeptolin A, B, and C and AER A and D), was found with no concomitant growth response (12). At the same time, P. agardhii, which suffered growth inhibition, smaller trichome size, and other alterations in cell morphology due to M. aeruginosa, produced fewer intracellular metabolites than in its monoculture. Nevertheless, like M. aeruginosa, P. agardhii produced a few specific compounds exclusively in response to the interactions (12). Therefore, it can be concluded that chemically mediated interspecific interactions promote the production of interaction-specific NRPs by cyanobacteria, regardless of their effect on the producer itself or the competitor. Nevertheless, to attest to this hypothesis, a three-step experiment should be conducted, where individual populations are first simulated in separate compartments, then in a shared medium allowing each population to take up substances secreted by the other, and finally in mixed culture.

The NRPs that significantly increased in the cocultures (i.e., SPU 599, AER 615, NOD 811, NOD 825, and AP 842) may have various roles in chemical communication and would be the most likely candidates as allelochemicals. However, to function as allelochemicals, they must be actively excreted by the cyanobacterium and not remain cell bound until the cell lysis (53, 54). Therefore, only NOD 825 and AP 842, which were detected extracellularly (H3; Fig. S5), can be considered putative allelochemicals, although other NRPs with potential allelochemical proprieties might be present extracellularly at concentrations below the limit of detection of the liquid chromatography-tandem mass spectrometry (LC-MS/MS) method used. Extracellular NOD 825 and AP 842 were present in the cocultures but not in the monoculture (Fig. S5), suggesting that presence of the algae induced the exudation, thus supporting the hypothesis H2. Moreover, the total concentrations of these NRPs were significantly higher in the cocultures than in the monocultures (Fig. 3 and Table S5), indicating a higher production in the presence of potential competitors. Accordingly, Møgelhøj et al. (19) detected a higher percentage of extracellular NOD in a mixed culture of N. spumigena and R. salina than in the N. spumigena monoculture. Thus, it is likely that infochemical sensing of potential competitors led to an upregulation of NOD 825 and AP 842 production and their subsequent exudation by N. spumigena. Jonasson et al. (55) suggested that NOD is released when its intracellular concentration reaches a certain threshold. In N. spumigena blooms, NODs are mostly cell bound (56), with up to 20% found extracellularly (57). Although more extracellular NODs are commonly observed due to the cell lysis (27), Hobson and Fallowfield (58) reported NOD release by intact cells. We have also observed an increase in cell-bound AP concentration and its release in the coculture of two strains of N. spumigena, belonging to different chemotype subgroups (11), which supports the potential involvement of APs in the cell‐to‐cell communication as a signaling molecule and a bioactive metabolite.

The NRPs that increased in the cocultures, but were not detected extracellularly, i.e., SPU 599, AER 615, and NOD 811 (Fig. S5 and Table S5), might have been produced to provide a competitive advantage to the cyanobacterium. However, no significant relations were observed between the algal growth and the upregulated NRPs in the cocultures (Table S6), suggesting that these NRPs (SPU 599, AER 615, NOD 811, NOD 825, and AP 842) had no allelopathic activity in our experimental conditions. It is also possible that some NRPs were not detected in the media because they quickly break down when released, especially considering that N. spumigena cultures were nonaxenic, and the associated cyanobacteria microbiome has been shown to degrade NRPs produced by the cyanobacterium (59). Moreover, our NRP concentrations based on the dry weight normalization might be higher than estimates based on the cell number if the cell masses in the rapidly growing strains are lower due to stress conditions (60).

Nevertheless, SPU 611 was significantly negatively related to P. tricornutum growth and significantly positively to R. salina growth (Table S6). In addition, the latter was inhibited by AP 828 (Table S6). Thus, although the main effect on the competitors was exerted by elevated pH, SPU 611 and AP 828 released by the cyanobacteria might be biologically active toward the algae, which can be addressed in future experimental studies.

### Synthesis of spumigins might be costly.

Considering that NRP production has been linked to optimal growth (26, 27), a positive relationship between the total NRP concentrations and N. spumigena growth was expected (H4). However, in N. spumigena mono- and cocultures, some spumigins (SPU 599 and SPU 641) were negatively related to the cyanobacteria growth (Fig. 5), suggesting that upregulation of their synthesis was suboptimal for the growth. Spumigins are potent serine protease inhibitors (61), which inhibit proteases that may be self-destructive (62). In Gram-negative and Gram-positive bacteria, serine proteases are membrane proteases required for processing newly synthesized secreted proteins, cell-cell adhesion, and surface colonization (63–65). Thus, as serine protease inhibitors, spumigins might constitute an important mechanism for regulating proteolytic activity in N. spumigena, such as the regulation of proteases involved in growth (66). Moreover, N. spumigena produces other SPUs that were unrelated to its growth (Fig. 5), including the total SPU concentration (Fig. 5 and Table S5). In cyanobacteria, SPUs might have multiple functions, but how their diversity is related to N. spumigena adaptability remains to be studied. Also, due to their common metabolic pathways and structural similarity, it can be speculated that different SPUs may have complementary functions as previously suggested (9–11). Thus, the upregulation of one SPU might compensate for downregulation of another.

### Conclusions.

In the coculture of N. spumigena with algal competitors, several NRPs significantly increased compared to the monoculture, and some were detected extracellularly, which coincided with growth inhibition in the cyanobacterium exposed to P. tricornutum. Although this diatom is known to produce allelochemicals suppressing various algae, this is the first report of such effects on cyanobacteria. In turn, the coculture with N. spumigena caused a significant extension of R. salina lag phase due to pH elevation, while P. tricornutum growth was stimulated at high pH. The NRP concentrations in the cocultures were significantly different from the N. spumigena monocultures, with a 5-fold NOD upregulation and exudation of NOD 825 and AP 842 in the cocultures. Thus, chemically mediated interspecific interactions induce the release of specific NRPs by N. spumigena, which can impinge on chemical interactions in plankton communities. Taken together, our findings suggest that it is not the specific allelopathic compounds but tolerance to alkaline conditions that conveys competitive advantage during the cyanobacteria blooms, although combined effects of pH and NRP produced by the cyanobacterium cannot be ruled out.

## MATERIALS AND METHODS

### Algal species and culture conditions.

The cyanobacterium Nodularia spumigena Mertens ex Bornet & Flahault (CCNP 1403) was isolated from the Baltic Sea and obtained from the Culture Collection of Northern Poland, Department of Marine Biotechnology, Gdańsk University, Poland. The diatom Phaeodactylum tricornutum Bohlin (CCAP 1052/1A) and the cryptomonad Rhodomonas salina (Wislouch) Hill & Wetherbee (CCAP 978/24) were obtained from the Culture Collection of Algae and Protozoa in Oban, United Kingdom (see Table S1 in the supplemental material). Although the diatom and the cryptomonad strains were not isolated from the Baltic Sea, these species are present in different Baltic Sea subbasins, and their distributions overlap N. spumigena (49). The cultures were maintained in 500-mL Erlenmeyer flasks containing 250 mL of f/2 medium (67), supplemented with NaCl to salinity 7 at 20 ± 2°C and irradiance of 30 μmol photons m^−2^·s^−1^ with a light/dark cycle of 16:8 h. Cultures were kept in the exponential-growth phase by repeated dilution in a fresh culture medium.

### Experimental setup.

**(i) Cocultivation system.** Monoculture and coculture exposures were conducted in the custom-made cocultivation system (Fig. S1) (11). In brief, the system consisted of two modified glass flasks, each holding 500 mL, fitted together by a holding clamp. The flasks were joined by a 0.22-μm hydrophilic polyvinylidene fluoride (PVDF) membrane filter (Durapore, Merck, Darmstadt, Germany) with free diffusion of dissolved substances, including NRPs, but not the algal cells. The NRP diffusion rate in this cocultivation system was demonstrated to be rapid, with the levels of NRPs dissolved in the media reaching equilibrium between the two coculture chambers after 10 h of the incubation (11).

**FIG 6:**
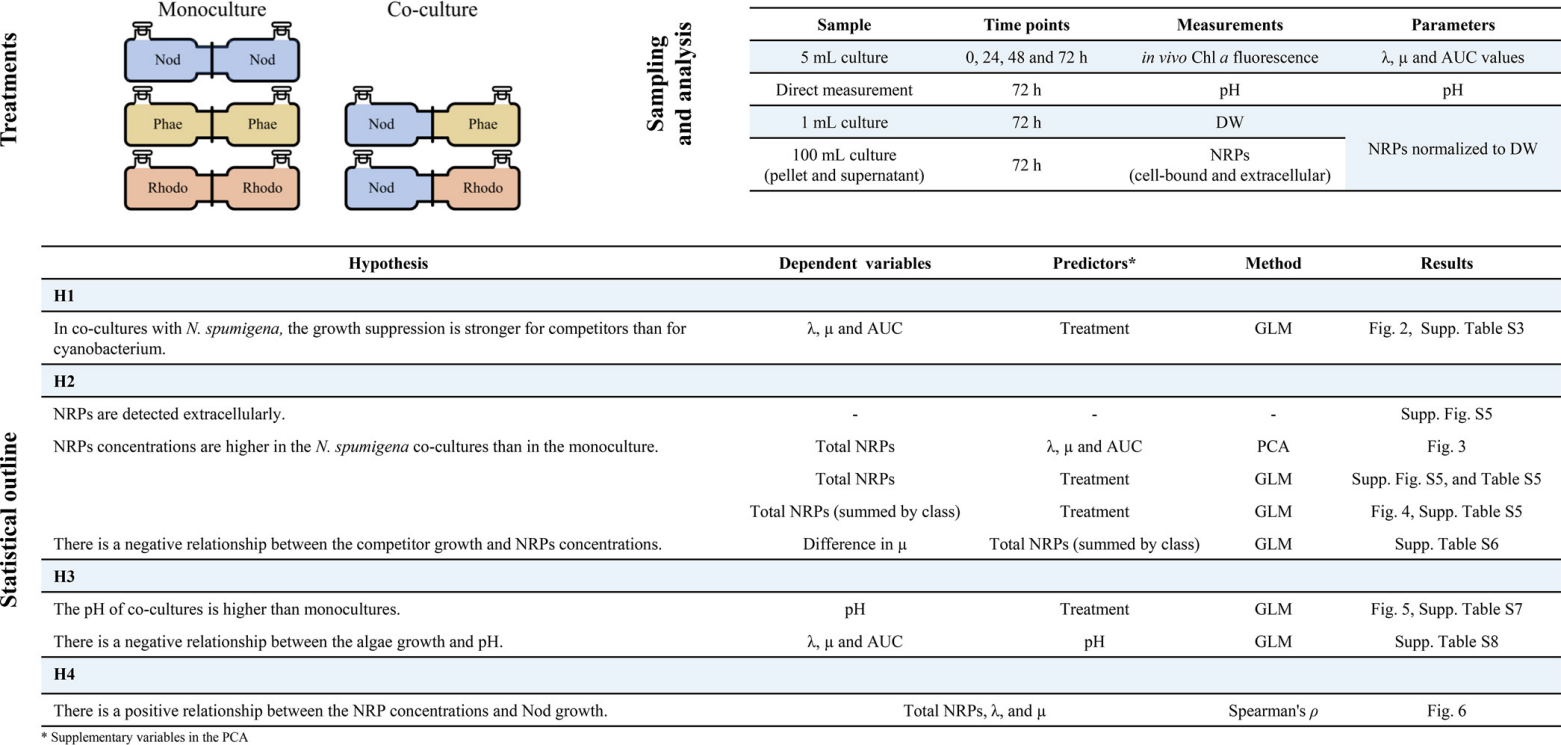
Schematic representation of the experimental design align with the hypotheses, research questions, and statistical methods used in the data analysis.

**(ii) Mono- and coculture experiments.** Exponentially growing cultures of the test species (N. spumigena, P. tricornutum, and R. salina) were diluted with f/2 media to the identical *in vivo* Chl*a* fluorescence values measured in triplicate using a 10-arbitrary unit (AU) field fluorometer (Turner Designs, Sunnyvale, CA, USA). In the monoculture treatments, both chambers were inoculated with 300 mL stock of the same species (either N. spumigena, P. tricornutum, or R. salina). In the cocultures (referred to as N. spumigena/P. tricornutum and N. spumigena/R. salina), 300 mL of N. spumigena were added to one of the chambers and 300 mL of either P. tricornutum or R. salina to the other chamber (Fig. 6). Each monoculture and coculture treatment was carried out in triplicate at the same experimental conditions as the inoculum cultures and shaken at approximately 50 rpm to prevent sedimentation and facilitate metabolite mixing in the system; the experiment lasted 72 h.

**(iii) Sampling and sample preparation.** Samples (5 mL) were collected at time points 0, 24, 48, and 72 h from all chambers to determine the *in vivo* Chl*a* fluorescence. At the end of the experiment (72 h), prior to N. spumigena, R. salina, and P. tricornutum biomass sample collection, pH was measured by introducing the pH electrode (PHM210; Radiometer Analytical, Loveland, CO, USA) on both chambers of each coculture system. pH was measured in triplicate in each monoculture/coculture. At the time point of 72 h, 100-mL samples were taken from all experimental units and vacuum filtered onto 47-mm GF/F filters (Whatman, Kent, UK). The wet filters with the retained cells were transferred to 2-mL Eppendorf tubes, dried under vacuum by rotary evaporation in an Eppendorf concentrator 5301 at room temperature (Eppendorf, Hamburg, Germany), and stored at −80°C until extraction. The supernatants of these samples were concentrated by the solid-phase extraction method using 500-mg Oasis HLB cartridges (Waters, Milford, MA, USA), according to Mazur-Marzec et al. (8). The 100% methanol eluate was evaporated to dryness following the same procedure as the filters and kept at −80°C until further workup. Additionally, 1-mL samples were collected at 72 h from all N. spumigena cultures and dried in the Eppendorf concentrator 5301 at 60°C. These samples were used to determine N. spumigena dry weight using a Sartorius BP211D analytical balance with a readability of 0.01 mg (Sartorius Lab Instruments GmbH & Co. KG, Göttingen, Germany).

### Growth assessment.

The measured time-specific fluorescence values at time points 0, 24, 48, and 72 h were used to model growth (68) with DMFit software (www.combase.cc) for each species/treatment/replicate. The model fit was assessed by the coefficient of determination (*R*^2^). The estimated parameters were the lag-phase duration (λ, h) and the maximal growth during the exponential phase (μ, day^−1^). The lag-phase duration shows how fast the test culture acclimates to the treatment conditions, while the maximal growth rate is the rate of increase during the exponential part of the growth curve. We also calculated the area under the curve (AUC) using the time-specific fluorescence after subtracting the initial fluorescence value as an additional growth parameter to represent time-integrated production. This endpoint is commonly used in algal growth inhibition assays (69), as it integrates the change in photosynthetically active biomass during exposure (70).

### Cell-bound and extracellular NRPs.

**(i) Extraction.** Before liquid chromatography-tandem mass spectrometry (LC-MS/MS) analysis, 1 mL 75% methanol in Milli-Q water was added to the dry filters (containing the N. spumigena, P. tricornutum, and R. salina cells, i.e., the cell-bound NRPs) and to the dried filtrate solid-phase extraction (SPE) eluates (containing extracellular NRPs). Filters were macerated with a fine glass rod. The samples (filters and filtrates) were homogenized in an ultrasonic bath (Sonorex; Bandelin, Berlin, Germany) for 5 min and vortexed for another 5 min. Subsequently, the samples were centrifuged for 10 min at 2°C and 10,000 rpm; each sample supernatant was transferred to chromatographic vials and analyzed by LC-MS/MS.

**(ii) LC-MS/MS analysis and quantification.** The LC-MS/MS analyses were performed according to Mazur-Marzec et al. (8) using an Agilent 1200 (Agilent Technologies, Waldbronn, Germany) coupled with a triple-quadrupole mass spectrometer (5500 QTrap; AB Sciex, Concord, ON, Canada) and a Zorbax Eclipse XDB-C_18_ column (4.6 mm by 150 mm; 5 μm; Agilent Technologies, Santa Clara, CA, USA) with a mobile phase composed of 5% acetonitrile in Milli-Q water (A) and acetonitrile (B), both containing 0.1% formic acid. The injection volume was 5 μL, and the flow rate was 0.6 mL min^−1^. The column temperature was 35°C. The gradient elution started with 15% of B, rising to 50% B over 10 min and then to 99% B in 5 min, held for 10 min, then decreased to 15% B in 2 min, and held for 10 min to equilibrate the system. Ionization was performed with electrospray (ESI) source in positive mode, with turbo ion spray (550°C) voltage of 5.5 kV and declustering potential of 80 V. MS detection was performed with the information-dependent acquisition (IDA) and enhanced ion product (EIP) modes. Detection of NRPs was performed in the *m/z* range of 500 to 1,000; when the signal of an ion was above a threshold of 500,000 cps, the EIP was automatically triggered, and the ions were fragmented in the collision cell (Q2) with collision energy (CE, 45 to 70 V) to optimize the achievement of the richest ion fragmentation spectrum. For NRP identification, the protonated ions’ *m/z*, their fragmentation spectra, and retention times were examined. The peak areas of the NRPs in the extracts were acquired from the extracted ion chromatograms of the NRPs’ protonated ions masses. Data acquisition and processing were accomplished using Analyst QS 1.5.1 software (AB Sciex, Concord, ON, Canada). The relative NRP concentrations in the extracts were based on the peak area of the ion chromatograms. Peak areas of cell-bound and extracellular NRPs were normalized to the dry mass of the N. spumigena sample taken at 72 h following the common practice and dividing the peak area by the dry mass (9, 71).

### Data analysis and statistics.

Statistical analyses were carried out with JMP version 14.0 (SAS Institute Inc., Cary, NC); the significance level was set to α of 0.05, and the data are presented as mean ± standard error (SE; *n *= 3 in all cases). See Fig. 6 for an overview of the statistical methods used to test the hypotheses. The variables included total NRP concentrations (i.e., normalized peak areas reflecting the sum of cell-bound and extracellular NRP quantities), growth parameters (λ, μ, and AUC), and pH values measured in each chamber of each exposure unit. The total NRP concentrations were grouped by class and expressed as the relative fold change between the normalized peak area in the cocultures and the respective monoculture. The fold change was calculated as *A*/*B*; with *A* being a replicate of a coculture NRP peak area and *B* the average monoculture peak area for the same NRP.

First, to explore the overall variability in the N. spumigena growth and NRP levels across the treatments, a principal-component analysis (PCA) on covariances was performed using the normalized total NRPs as response variables and the growth parameters (λ, μ, and AUC) as supplementary variables. The explained proportion of the variance for each component and the minimum number of the components were determined by the broken stick model (Fig. S2). As only one significant PC was identified (Table S2 and Fig. S3), the regression analysis was applied to identify significant predictors.

Second, to evaluate interactions between N. spumigena and the competitors (hypothesis H1), generalized linear models (GLM) with normal error structure and identity link were used. No data transformation was needed for the variables λ, μ, AUC, and pH, whereas the NRP values were log transformed. The residual analysis and Q-Q plots were used to examine the goodness of fit and homoscedasticity of the models. For each species, the treatment (monoculture versus coculture) effect on each growth parameter (λ, μ, and AUC values) was evaluated to address hypothesis H1.

In addition to the models for the growth parameters, we evaluated the relationship between the maximal growth rate and lag-phase duration across the treatments in all species. A positive relation between these parameters has been used to indicate dynamic adaptation to the growth conditions and compensatory growth following a prolonged lag phase (30). To address hypothesis H2, a GLM was used to examine treatment effects on the NRP levels in N. spumigena. Further, to evaluate the relationship between the growth and total NRPs in each competitor (Phae and Rhodo), we used a ratio for the maximal growth rate between its cocultures and the monocultures as a dependent variable and total NRP concentrations as predictors. Although only a few NRPs were detected extracellularly, these GLMs were performed for all NRPs. We used the Spearman rank correlation to evaluate relations between NRP levels and growth parameters in N. spumigena (hypothesis H4). Finally, a set of GLMs was used to evaluate whether (i) pH was affected by the treatment (monoculture versus coculture in N. spumigena, Phae, and Rhodo), and (ii) growth parameters were affected by the pH in all species (hypothesis H3).
