# Supplementary material for: Interspecific Interactions Drive Nonribosomal Peptide Production in Nodularia spumigena
Source: Appl Environ Microbiol. 2022 Jul 12;88(15):e00966-22. doi: 10.1128/aem.00966-22 (PMC9361812; doi:10.1128/aem.00966-22)
Supplement: Supplemental file 1 — Fig. S1 to S5 and Tables S1 to S8. Download aem.00966-22-s0001.pdf, PDF file, 0.4 MB [file aem.00966-22-s0001.pdf]

Supplementary Material

Interspecific interactions drive non-ribosomal peptide production in *Nodularia spumigena*

Sandra Lage[1,2,*], Hanna Mazur-Marzec[2], Elena Gorokhova[1,*]

[1] Department of Environmental Science, Stockholm University, Sweden

[2] Division of Marine Biotechnology, Institute of Oceanography, University of Gdańsk, Poland

*Correspondence: smlage@ualg.pt; elena.gorokhova@aces.su.se

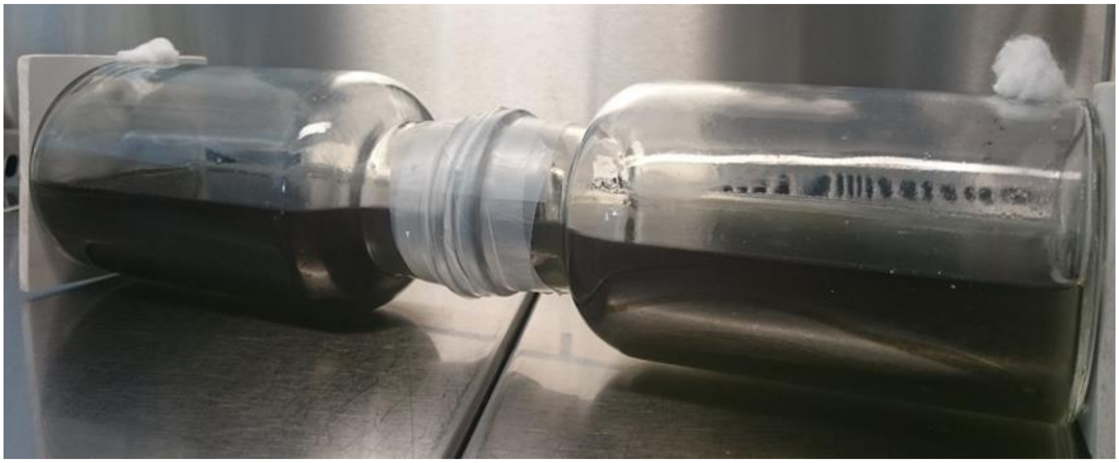

Supplementary Figure S1. The co-culture system consisting of two modified glass flasks each holding 500 mL fitted together by a holding clamp. The two chambers are divided by a 0.22 μm hydrophilic polyvinylidene fluoride (PVDF) membrane filter that enabled the passage of dissolved substances but not cells. All components of the co-culture system were autoclaved separately and assembled under sterile conditions. The photo was taken by Sandra Lage.

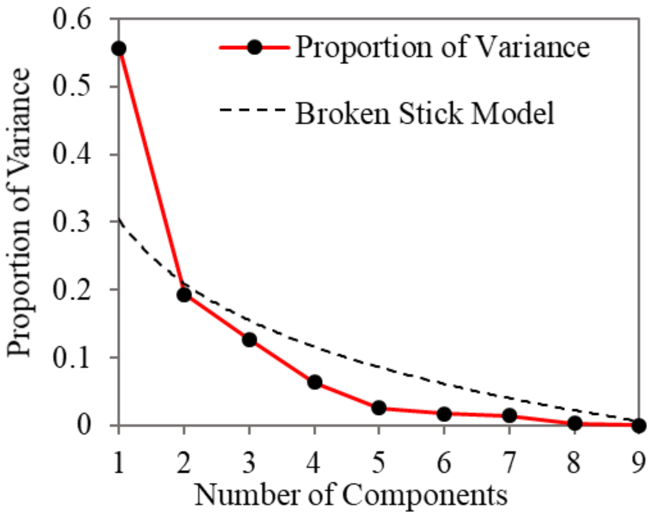

Supplementary Figure S2. Scree plot showing the proportion of variance explained by principal components using the sum of cell-bound and extracellular NRPs of *N. spumigena* in monocultures and co-cultures with *P. tricornutum* and *R. salina*. The dashed curve indicates the expected proportions estimated by the broken stick model and the significant principal components (PCs). The significance of the components was obtained by comparing the explained proportion of variance (red line) and the minimum proportion of variance expectation under the broken stick model (dashed line). The broken-stick model test shows one significant PC, suggesting that the relationship can be described by a univariate regression.

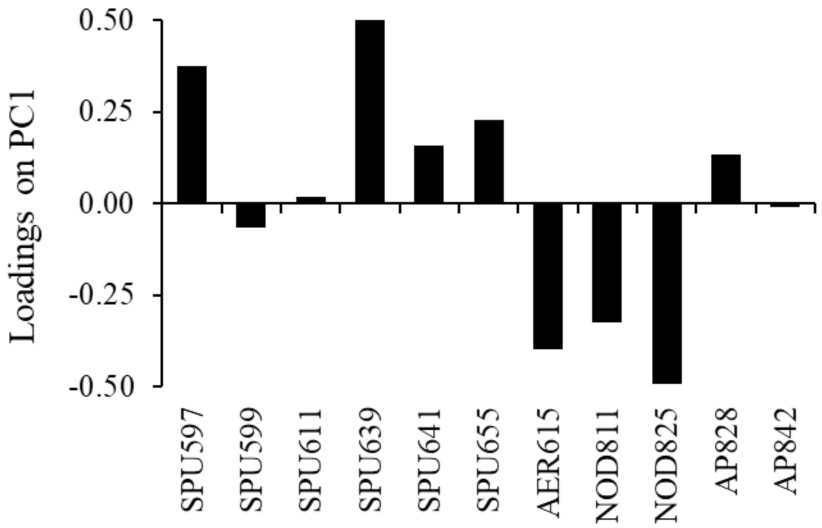

Supplementary Figure S3. Contribution of different NRPs of *N. spumigena* in monocultures and co-cultures with *P. tricornutum* and *R. salina* to the PC1. The spumigins (SPU 597 and SPU 639) had the highest positive loadings (≥0.3), whereas the aeruginosin (AER 615) and the nodularins (NOD 811 and NOD 825) had the highest negative loadings on the PC1, and, thus, the highest influence on the grouping.

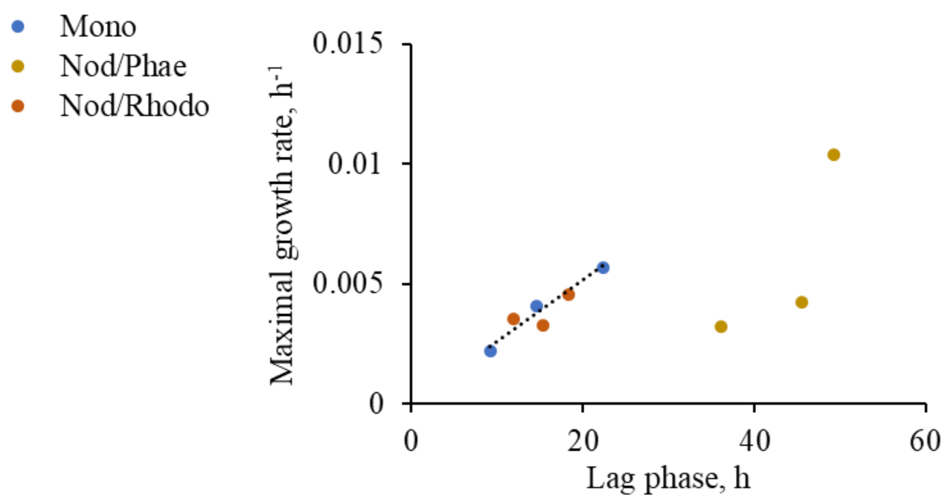

Supplementary Figure S4. Relationship between the maximal growth rate (μ, h^{-1}) and duration of the lag phase (λ, h) in *N. spumigena* monoculture and co-culture with *P. tricornutum* (Nod/Phae) and *R. salina* (Nod/Rhodo); see Table S4 for the regression output.

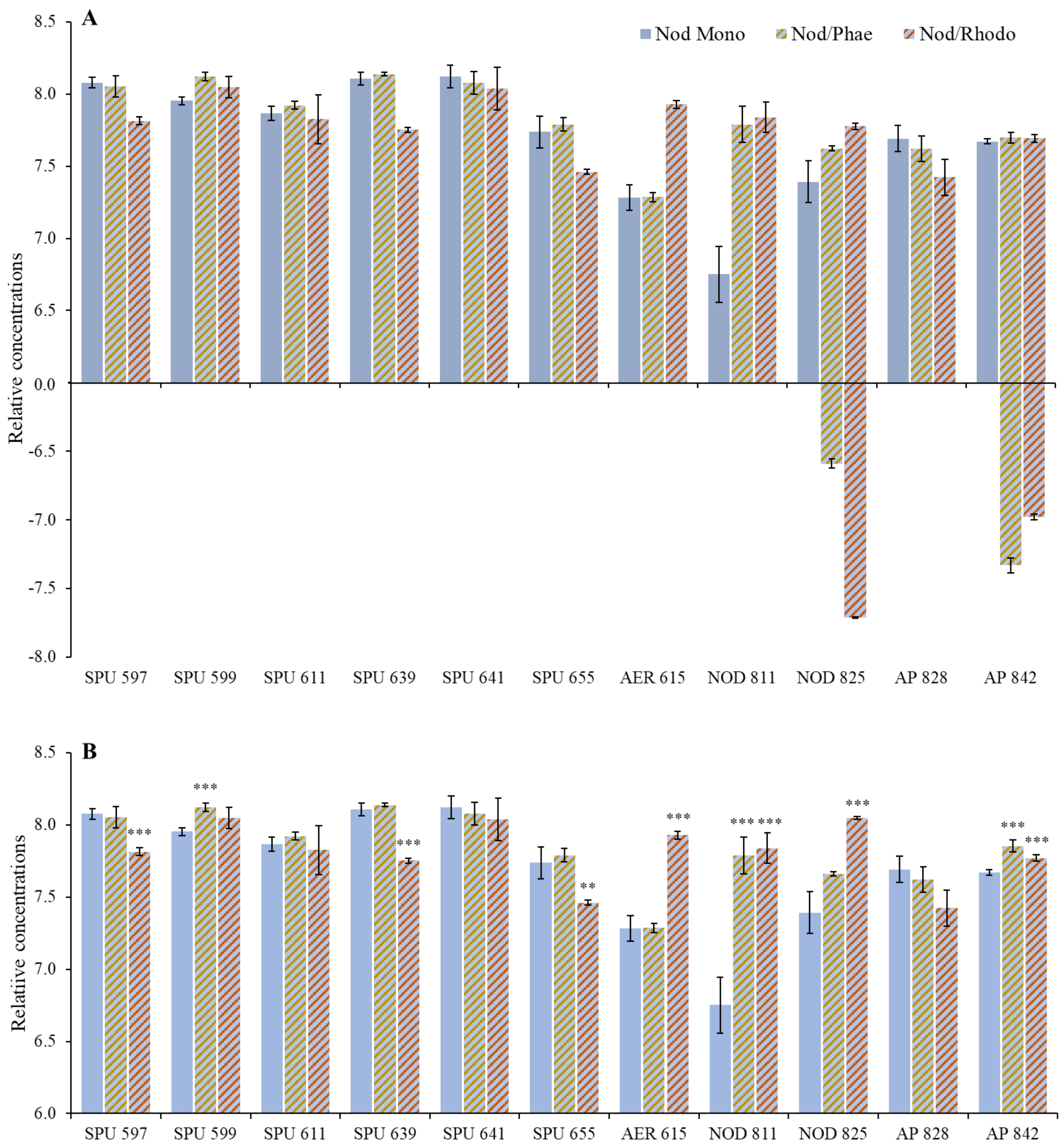

Supplementary Figure S5. Relative concentrations of (A) cell-bound and extracellular NRPs and (B) total (cell-bound and extracellular) NRPs in the *N. spumigena* monoculture (Mono) and co-cultures with *P. tricornutum* (Nod/Phae) and *R. salina* (Nod/Rhodo). The data are shown as log-transformed peak areas normalized to the dry weight (mean ± standard error, n = 3). Cell-bound and extracellular NRPs are plotted as positive and negative values, respectively. NRPs are denoted as SPU, spumigin; NOD, nodularin; AER, aeruginosin; AP, anabaenopeptin. Monocultures are shown with solid colors and co-cultures are shown in stripe patterns. Asterisk (*) indicates a significant difference from the monoculture; *: $p < 0.05$, **: $p < 0.01$, and ***: $p < 0.001$ (GLM; Supplementary Table S5).

Supplementary Table S1. Description of *Nodularia spumigena*, *Phaeodactylum tricornutum* and *Rhodomonas salina* strains used. CCNP: Culture Collection of Northern Poland, Dept. of Marine Biotechnology, Gdańsk University, Poland; CCAP: Culture Collection of Algae and Protozoa, Scottish Association for Marine Science, Oban, UK. DNA sequences can be retrieved from GenBank.

Species	Strain Code	Area of Isolation	Isolation Year	GenBank Accesssion Number
N. spumigena	CCNP1403	Gulf of Gdańsk (54°29′ N, 18°40′ E)	1997	KC788205
P. tricornutum	CCAP 1052/1A	Off Plymouth, Devon, England, UK	1990	DQ085802<br>DQ402479<br>FR865502
R. salina	CCAP 978/24	St. Helens River, Bembridge, Isle of Wight, England, UK	1959	EU926158<br>FJ210726

Supplementary Table S2. Principal Components (PC), eigenvalues and % of variance, and cumulative (Cum.) % of variance explained by the PCs of the PCA of the cell-bound and extracellular NRPs of *N. spumigena* monocultures and co-cultures with *P. tricornutum* and *R. salina*.

PC	Eigenvalue	% Variance	Cum. % Variance
1	5.83×10^{15}	55.710	55.710
2	2.03×10^{15}	19.356	75.066
3	1.32×10^{15}	12.652	87.718
4	6.63×10^{14}	6.334	94.052
5	2.67×10^{14}	2.551	96.603
6	1.77×10^{14}	1.695	98.298
7	1.49×10^{14}	1.420	99.718
8	2.95×10^{13}	0.282	100
9	0.566	0.000	100

Supplementary Table S3. GLM outcome on treatment effect (monoculture *vs.* co-culture) on the growth parameters; lag phase duration (λ, h), maximal growth during exponential phase (μ, h^{-1}), and area under curve (AUC) of *N. spumigena* (Nod), *P. tricornutum* (Phae) and *R. salina* (Rhodo) mono- and co-cultures. Significant differences from the monoculture are in bold; monoculture was selected as reference group.

Species	Dependent variable	Co-culture	Estimate	Std. error	Wald Stat.	*p-value*
Nod	λ	Nod/Phae	28.248	5.482	26.548	**<0.0001**
		Nod/Rhodo	-0.250	4.243	0.004	0.953
	μ	Nod/Phae	0.002	0.002	0.648	0.421
		Nod/Rhodo	-1.833×10^{-4}	1.064×10^{-3}	0.030	0.863
	AUC	Nod/Phae	-14.300	6.113	5.472	**0.019**
		Nod/Rhodo	2.628	4.712	0.311	0.577
Phae	λ	Nod/Phae	1.114	0.773	2.079	0.149
	μ	Nod/Phae	0.004	0.001	9.542	**0.002**
	AUC	Nod/Phae	1.960	7.294	0.072	0.788
Rhodo	λ	Nod/Rhodo	11.104	2.640	17.691	**<0.0001**
	μ	Nod/Rhodo	-1.167×10^{-5}	2.051×10^{-4}	3.235×10^{-3}	0.955
	AUC	Nod/Rhodo	-12.280	15.740	0.608	0.435

Supplementary Table S4. GLM for maximal growth (μ, h^{-1}) as a function of lag phase duration (λ, h) in *N. spumigena* (Nod), *P. tricornutum* (Phae), and *R. salina* (Rhodo) mono- and co-cultures. Significant effects ($p < 0.05$) are in bold, and marginally significant ($p < 0.1$) are in Italics.

Species	Treatment	Estimate	Std. error	Wald Stat.	*p-value*
Nod	Mono	2.58×10^{-4}	2.06×10^{-5}	11.931	**0.001**
	Nod/Phae	1.47×10^{-4}	8.22×10^{-5}	2.181	0.140
	Nod/Rhodo	4.56×10^{-4}	1.98×10^{-4}	3.049	*0.081*
Phae	Mono	8.82×10^{-4}	7.15×10^{-5}	11.832	**0.001**
	Nod/Phae	-5.30×10^{-4}	2.212×10^{-3}	0.057	0.811
Rhodo	Mono	6.60×10^{-5}	3.9×10^{-5}	2.008	0.157
	Nod/Rhodo	-6.75×10^{-5}	3.68×10^{-4}	0.033	0.855

Supplementary Table S5. GLM outcome for treatment effects (monoculture *vs.* co-culture) on the total NRPs (cell-bound and extracellular, individual and aggregated by class) produced by *N. spumigena* (Nod) in co-culture with *P. tricornutum* (Phae) and *R. salina* (Rhodo). NRPs are denoted as SPU, spumigins; NOD, nodularins; AER, aeruginosins; AP, anabaenopeptins. Monoculture was selected as a reference group; significant differences from monoculture are in bold.

Dependent variable	Co-culture	Estimate	Std. error	Wald Stat.	*p-value*
SPU 597	Nod/Phae	-0.024	0.081	0.090	0.765
	Nod/Rhodo	-0.264	0.045	33.868	**<0.0001**
SPU 599	Nod/Phae	0.169	0.041	16.962	**<0.0001**
	Nod/Rhodo	0.095	0.079	1.426	0.233
SPU 611	Nod/Phae	0.057	0.055	1.065	0.301
	Nod/Rhodo	-0.039	0.175	0.050	0.823
SPU 639	Nod/Phae	0.030	0.047	0.412	0.521
	Nod/Rhodo	-0.354	0.048	53.847	**<0.0001**
SPU 641	Nod/Phae	-0.044	0.110	0.160	0.689
	Nod/Rhodo	-0.083	0.166	0.247	0.619
SPU 655	Nod/Phae	0.052	0.119	0.195	0.658
	Nod/Rhodo	-0.276	0.111	6.203	**0.013**
SPU	Nod/Phae	0.240	0.373	0.416	0.519
	Nod/Rhodo	-0.921	0.341	7.314	**0.007**
AER 615 / AER	Nod/Phae	4.954×10^{-3}	0.094	2.778×10^{-3}	0.958
	Nod/Rhodo	0.646	0.093	48.562	**<0.0001**
NOD 811	Nod/Phae	1.039	0.231	20.131	**<0.0001**
	Nod/Rhodo	1.088	0.220	24.420	**<0.0001**
NOD 825	Nod/Phae	0.270	0.145	3.428	0.064
	Nod/Rhodo	0.655	0.145	20.333	**<0.0001**
NOD	Nod/Phae	1.309	0.318	16.916	**<0.0001**
	Nod/Rhodo	1.743	0.307	32.278	**<0.0001**
AP 828	Nod/Phae	-0.072	0.126	0.328	0.567
	Nod/Rhodo	-0.269	0.153	3.073	0.080
AP 842	Nod/Phae	0.181	0.045	16.052	**<0.0001**
	Nod/Rhodo	0.098	0.028	12.490	**0.0004**
AP	Nod/Phae	0.109	0.137	0.638	0.425
	Nod/Rhodo	-0.170	0.158	1.167	0.280

Supplementary Table S6. GLM outcome for NRPs produced by *N. spumigena* effects on the ratio co-culture/monoculture of the maximal growth during exponential phase (μ, h^{-1}) of *P. tricornutum* (Phae) and *R. salina* (Rhodo). NRPs are denoted as SPU, spumigins; NOD, nodularins; AER, aeruginosins; AP, anabaenopeptins. Significant effects are in bold; (*) indicates NRPs detected extracellularly.

Species	Predictors	Estimate	Std. error	Wald Stat.	*p*-value
Phae	SPU 597	0.314	1.548	0.041	0.839
	SPU 599	-1.257	3.703	0.115	0.734
	SPU 611	-3.991	1.704	5.489	**0.019**
	SPU 639	-7.339	4.621	2.522	0.112
	SPU 641	-0.990	1.071	0.854	0.355
	SPU 655	-1.962	1.471	1.778	0.182
	AER 615	-2.598	2.405	1.167	0.280
	NOD 811	0.769	0.462	2.763	0.097
	NOD 825*	4.554	6.647	0.469	0.493
	AP 828	1.079	0.729	2.191	0.139
	AP 842*	0.694	2.690	0.067	0.796
Rhodo	SPU 597	2.004	1.029	3.792	0.052
	SPU 599	0.477	0.646	0.547	0.460
	SPU 611	0.344	0.077	20.054	**<0.0001**
	SPU 639	0.900	3.315	0.074	0.786
	SPU 641	-0.163	0.370	0.195	0.659
	SPU 655	0.452	3.172	0.020	0.887
	AER 615	-0.417	2.057	0.041	0.839
	NOD 811	0.357	0.440	0.659	0.417
	NOD 825*	0.516	5.451	0.009	0.925
	AP 828	-0.476	0.059	64.671	**<0.0001**
	AP 842*	1.834	2.268	0.654	0.419

Supplementary Table S7. GLM outcome for treatment (monoculture *vs*. co-culture) effect on the pH values in mono- and co-cultures of *N. spumigena* (Nod), *P. tricornutum* (Phae), and *R. salina* (Rhodo). Monoculture was used as a reference group; significant differences from the monoculture are in bold. See Fig. 5 for visualization of the results.

Species	Co-culture	Estimate	Std. error	Wald Stat.	*p-value*
Nod	Nod/Phae	0.113	0.165	0.468	0.494
	Nod/Rhodo	0.013	0.173	5.944×10^{-3}	0.939
Phae	Nod/Phae	0.620	0.133	21.569	**<0.0001**
Rhodo	Nod/Rhodo	0.513	0.127	16.423	**<0.0001**

Supplementary Table S8. GLM testing pH effects on the growth parameters in the mono- and co-cultures of *N. spumigena* (Nod), *P. tricornutum* (Phae), and *R. salina* (Rhodo). The growth parameters are lag phase duration (λ, h), maximal growth rate (μ, h^{-1}), and area under the curve (AUC). Significant effects ($p < 0.05$) are in bold, and marginally significant ($p < 0.1$) are in Italics

Species	Dependent variable	Estimate	Std. error	Wald Stat.	*p-value*
Nod	λ	-14.066	36.732	0.147	0.702
	μ	-7.980×10^{-4}	5.883×10^{-3}	0.018	0.892
	AUC	-5.612	23.399	0.057	0.811
Phae	λ	0.912	1.336	0.467	0.495
	μ	4.588×10^{-3}	2.352×10^{-3}	3.805	*0.051*
	AUC	4.059	10.711	0.144	0.705
Rhodo	λ	12.240	6.401	7.243	**0.007**
	μ	2.855×10^{-4}	8.661×10^{-4}	0.109	0.742
	AUC	-26.523	26.367	1.012	0.315
